# 1,2-Bis(2,4-di­nitro­phen­yl)disulfane

**DOI:** 10.1107/S1600536813011082

**Published:** 2013-04-30

**Authors:** Selvarasu Muthulakshmi, Doraisamyraja Kalaivani

**Affiliations:** a PG and Research Department of Chemistry, Seethalakshmi Ramaswami College, Tiruchirappalli 620 002, Tamil Nadu, India

## Abstract

In the title mol­ecule, C_12_H_6_N_4_O_8_S_2_, the dihedral angle between the benzene rings is 77.00 (8)°. The mean planes of the nitro groups are twisted slightly from the benzene rings, forming dihedral angles in the range 2.3 (2)–8.6 (3)°. The S—S bond length is 2.0458 (7) Å. Each S atom is essentially coplanar with the benzene ring to which it is attached, with deviations from the ring planes of 0.0163 (5) and 0.0538 (5) Å. In the crystal, mol­ecules are linked through weak C—H⋯O hydrogen bonds, forming a two-dimensional network parallel to (001).

## Related literature

For synthetic applications of di­sulfides, see: Khavasch *et al.* (1950 ▶); Mitin & Zaperalova (1974 ▶); Stepanov *et al.* (1974 ▶, 1977 ▶); Cochran *et al.* (1996 ▶). For the natural occurrence of di­sulfides, see: Ramadas & Srinivasan (1995 ▶). For the preparation procedures for di­sulfides, see: Khavasch & Cameron (1951 ▶); Traynelis & Rieck (1973 ▶); Bilozor & Boldyrev (1984 ▶). For standard bond lengths, see: Allen *et al.* (1987 ▶). For related structures, see: Glidewell *et al.* (2000 ▶); Song & Fan (2009 ▶); Xiao *et al.* (2010 ▶); Buvaneswari *et al.* (2012 ▶). For hydrogen-bond graph-set motifs, see: Bernstein *et al.* (1995 ▶).
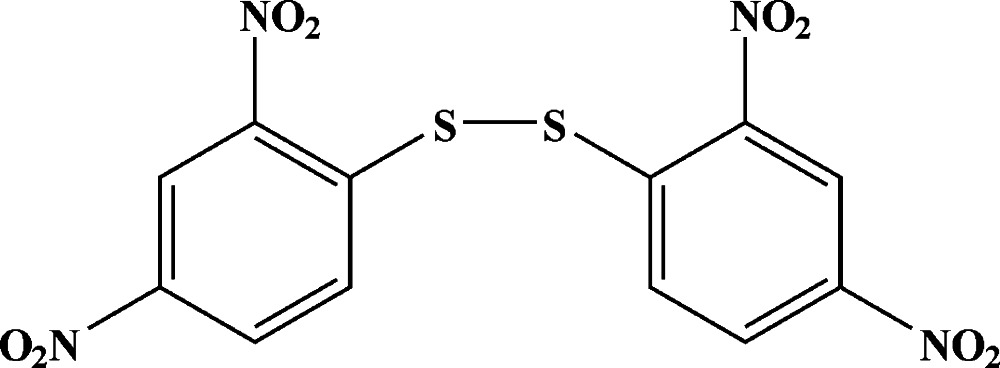


## Experimental

### 

#### Crystal data

C_12_H_6_N_4_O_8_S_2_
*M* *_r_* = 398.33
Monoclinic, 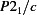

*a* = 11.3776 (6) Å
*b* = 11.9579 (5) Å
*c* = 11.0459 (6) Å
β = 90.943 (2)°
*V* = 1502.62 (13) Å^3^
*Z* = 4
Mo *K*α radiation
μ = 0.41 mm^−1^
*T* = 293 K
0.25 × 0.20 × 0.20 mm

#### Data collection

Bruker Kappa APEXII CCD diffractometer
Absorption correction: multi-scan (*SADABS*; Bruker, 2004 ▶) *T*_min_ = 0.804, *T*_max_ = 0.922
22589 measured reflections
5706 independent reflections
3983 reflections with *I* > 2σ(*I*)
*R*_int_ = 0.025

#### Refinement

*R*[*F* ^2^ > 2σ(*F* ^2^)] = 0.047
*wR*(*F* ^2^) = 0.141
*S* = 1.02
5706 reflections
235 parameters
H-atom parameters constrained
Δρ_max_ = 0.44 e Å^−3^
Δρ_min_ = −0.25 e Å^−3^

### 

Data collection: *APEX2* (Bruker, 2004 ▶); cell refinement: *APEX2* and *SAINT* (Bruker, 2004 ▶); data reduction: *SAINT* and *XPREP* (Bruker, 2004 ▶); program(s) used to solve structure: *SIR92* (Altomare *et al.*, 1993 ▶); program(s) used to refine structure: *SHELXL97* (Sheldrick, 2008 ▶); molecular graphics: *ORTEP-3 for Windows* (Farrugia, 2012 ▶) and *Mercury* (Macrae *et al.*, 2008 ▶); software used to prepare material for publication: *SHELXL97*.

## Supplementary Material

Crystal structure: contains datablock(s) global, I. DOI: 10.1107/S1600536813011082/lh5606sup1.cif

Structure factors: contains datablock(s) I. DOI: 10.1107/S1600536813011082/lh5606Isup2.hkl

Supplementary material file. DOI: 10.1107/S1600536813011082/lh5606Isup3.cml

Additional supplementary materials:  crystallographic information; 3D view; checkCIF report

## Figures and Tables

**Table 1:** Hydrogen-bond geometry (Å, °)

*D*—H⋯*A*	*D*—H	H⋯*A*	*D*⋯*A*	*D*—H⋯*A*
C5—H5⋯O2^i^	0.93	2.54	3.341 (3)	144
C8—H8⋯O3^ii^	0.93	2.60	3.403 (2)	144
C12—H12⋯O6^iii^	0.93	2.42	3.139 (2)	134

Symmetry codes: (i) 
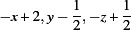
; (ii) 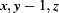; (iii) 
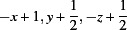
.

**Fig. 1.**
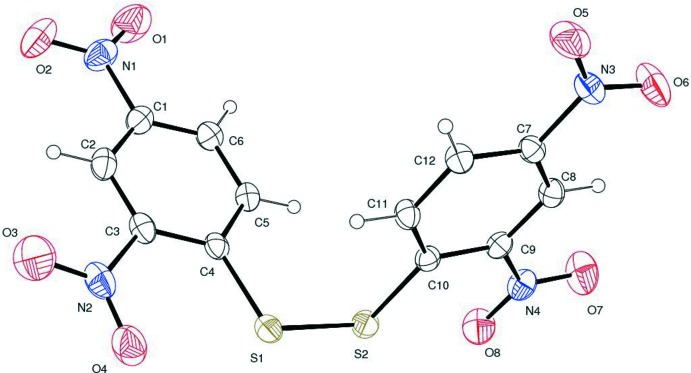
The molecular structure of the title conpound showing 30% probability displacement ellipsoids.

**Fig. 2.**
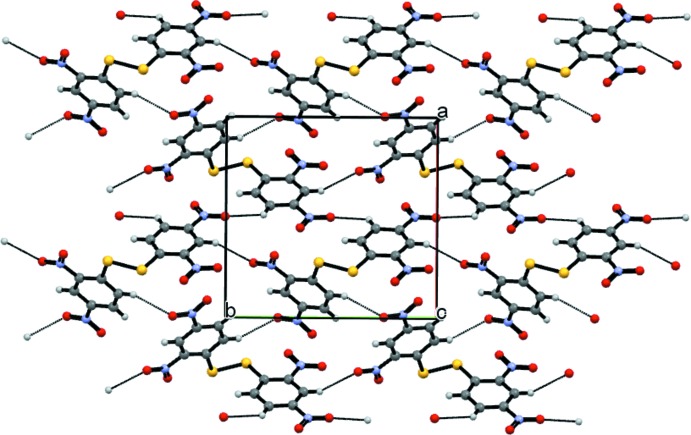
Part of the crystal structure with hydrogen bonds shown as dashed lines.

## supplementary crystallographic information

### Comment

The title molecule has acquired significance as it is employed to prepare several other synthetically important molecules (Khavasch *et al.*,1950; Stepanov *et al.*,1974; Stepanov *et al.*,1977; Cochran *et al.*,1996; Mitin & Zaperalova, 1974). Disulfide compounds are found in many naturally occuring compounds (Ramadas & Srinivasan, 1995). Despite the fact that several synthetic procedures are available for the preparation of title molecule, the yield of it is less than 55% in many cases (Khavasch & Cameron, 1951; Traynelis & Rieck, 1973; Bilozor & Boldyrev, 1984). In the present work, it is obtained in good yield (greater than 90%) with high purity through a one pot synthesis.

The molecular structure of the title compound is shown in Fig. 1. The bond lengths (Allen *et al.*, 1987) and bond angles are within normal ranges and are essentially the same in both chemically similar halves of the molecule. The the S—S bond is formally a single bond [S1—S2 bond length = 2.0458 (7)Å]. The dihedral angle between the benzene rings is 77.00 (8)°. Similar observations have been reported in related molecular structures (Glidewell *et al.*, 2000; Song & Fan, 2009; Xiao *et al.*, 2010; Buvaneswari *et al.*, 2012). The mean planes of the nitro groups are slightly twisted from the benzene rings forming dihedral angles of 4.4 (2), 8.6 (3), 5.3 (2) and 2.3 (2)° for the nitro groups containing N1, N2, N3 and N4 respectively. In the crystal, weak C—H···O hydrogen bonds (Table 1) connect molecules to form *R*_3_^3^(20) and *R*_3_^3^(22) graph-set motifs (Bernstein *et al.*, 1995) contained within two-dimensional corrugated sheets running parallel to (001) (Fig 2).

### Experimental

1-Chloro-2,4-dinitrobenzene (2 g, 0.01 mol) was dissolved in 20 ml of DMSO. Thiourea (0.8 g, 0.01 mol) was also dissolved in 20 ml of DMSO. These two solutions were mixed together and stirred well for about 2 hours and then allowed to stand 303K. On standing, a crystalline yellow solid separated out. The yellow coloured crystals were filtered and dried. The solid obtained was ground well and washed repeatedly with water, alcohol and ether to remove unreacted 1-Chloro-2,4-dinitrobenzene (DNCB) and thiourea (TU). The washed sample was recrystallised from acetic acid to yield single crystals. The yield of the pure compound was 95% (melting point greater than 533K). Micro analysis, calcd:C, 36.18; H,1.50; N, 14.07; found : C, 36.37; H, 1.27; N, 14.13. It is worth mentioning that in the reported preparation, the title molecule crystallizes in the pure form from the reaction mixture and the IR, PMR and micro analysis data of the sample before and after recrystallisation are exactly the same.

### Refinement

H atoms were placed in calculated positions with C—H = 0.93Å and were included in the refinement with U_iso_(H) = 1.2U_eq_(C).

### Crystal data

**Table:**

C_12_H_6_N_4_O_8_S_2_	*F*(000) = 808
*M**_r_* = 398.33	*D*_x_ = 1.761 Mg m^−^^3^
Monoclinic, *P*2_1_/*c*	Mo *K*α radiation, λ = 0.71073 Å
Hall symbol: -P 2ybc	Cell parameters from 7792 reflections
*a* = 11.3776 (6) Å	θ = 2.5–30.2°
*b* = 11.9579 (5) Å	µ = 0.41 mm^−^^1^
*c* = 11.0459 (6) Å	*T* = 293 K
β = 90.943 (2)°	Block, yellow
*V* = 1502.62 (13) Å^3^	0.25 × 0.20 × 0.20 mm
*Z* = 4	

### Data collection

**Table:**

Bruker Kappa APEXII CCD diffractometer	5706 independent reflections
Radiation source: fine-focus sealed tube	3983 reflections with *I* > 2σ(*I*)
Graphite monochromator	*R*_int_ = 0.025
ω and φ scan	θ_max_ = 33.5°, θ_min_ = 2.5°
Absorption correction: multi-scan (*SADABS*; Bruker, 2004)	*h* = −17→16
*T*_min_ = 0.804, *T*_max_ = 0.922	*k* = −17→18
22589 measured reflections	*l* = −16→16

### Refinement

**Table:**

Refinement on *F*^2^	Primary atom site location: structure-invariant direct methods
Least-squares matrix: full	Secondary atom site location: difference Fourier map
*R*[*F*^2^ > 2σ(*F*^2^)] = 0.047	Hydrogen site location: inferred from neighbouring sites
*wR*(*F*^2^) = 0.141	H-atom parameters constrained
*S* = 1.02	*w* = 1/[σ^2^(*F*_o_^2^) + (0.0681*P*)^2^ + 0.4377*P*] where *P* = (*F*_o_^2^ + 2*F*_c_^2^)/3
5706 reflections	(Δ/σ)_max_ < 0.001
235 parameters	Δρ_max_ = 0.44 e Å^−^^3^
0 restraints	Δρ_min_ = −0.25 e Å^−^^3^

### Special details

**Table:**

Geometry. All esds (except the esd in the dihedral angle between two l.s. planes) are estimated using the full covariance matrix. The cell esds are taken into account individually in the estimation of esds in distances, angles and torsion angles; correlations between esds in cell parameters are only used when they are defined by crystal symmetry. An approximate (isotropic) treatment of cell esds is used for estimating esds involving l.s. planes.
Refinement. Refinement of F^2^ against ALL reflections. The weighted R-factor wR and goodness of fit S are based on F^2^, conventional R-factors R are based on F, with F set to zero for negative F^2^. The threshold expression of F^2^ > 2sigma(F^2^) is used only for calculating R-factors(gt) etc. and is not relevant to the choice of reflections for refinement. R-factors based on F^2^ are statistically about twice as large as those based on F, and R- factors based on ALL data will be even larger.

### Fractional atomic coordinates and isotropic or equivalent isotropic displacement parameters (Å^2^)

**Table:**

	*x*	*y*	*z*	*U*_iso_*/*U*_eq_	
C1	0.94644 (14)	0.13448 (15)	0.26049 (15)	0.0423 (3)	
C2	0.87515 (16)	0.21656 (14)	0.21366 (17)	0.0459 (4)	
H2	0.8704	0.2863	0.2504	0.055*	
C3	0.81090 (15)	0.19244 (13)	0.11069 (16)	0.0414 (3)	
C4	0.81370 (13)	0.08818 (12)	0.05402 (14)	0.0360 (3)	
C5	0.88741 (14)	0.00751 (13)	0.10614 (15)	0.0402 (3)	
H5	0.8917	−0.0631	0.0711	0.048*	
C6	0.95385 (14)	0.03052 (14)	0.20845 (16)	0.0423 (3)	
H6	1.0032	−0.0237	0.2419	0.051*	
C7	0.56165 (13)	−0.32356 (13)	0.13396 (14)	0.0357 (3)	
C8	0.62734 (13)	−0.37316 (13)	0.04595 (15)	0.0386 (3)	
H8	0.6302	−0.4505	0.0380	0.046*	
C9	0.68881 (13)	−0.30433 (13)	−0.03018 (14)	0.0358 (3)	
C10	0.68625 (12)	−0.18758 (13)	−0.02111 (13)	0.0342 (3)	
C11	0.61794 (14)	−0.14233 (13)	0.07059 (15)	0.0391 (3)	
H11	0.6145	−0.0651	0.0798	0.047*	
C12	0.55557 (14)	−0.20961 (13)	0.14776 (15)	0.0385 (3)	
H12	0.5101	−0.1784	0.2082	0.046*	
N1	1.01562 (15)	0.15860 (17)	0.37076 (16)	0.0594 (4)	
N2	0.73842 (17)	0.28314 (13)	0.06113 (19)	0.0586 (4)	
N3	0.49685 (13)	−0.39543 (13)	0.21674 (14)	0.0477 (3)	
N4	0.75847 (13)	−0.36018 (14)	−0.12208 (14)	0.0476 (3)	
O1	1.08349 (18)	0.0867 (2)	0.40606 (18)	0.0917 (6)	
O2	1.00333 (17)	0.24936 (18)	0.41926 (17)	0.0815 (5)	
O3	0.7298 (2)	0.36795 (16)	0.1202 (2)	0.1180 (9)	
O4	0.69201 (16)	0.26986 (13)	−0.03687 (19)	0.0752 (5)	
O5	0.44650 (15)	−0.35239 (14)	0.30093 (15)	0.0700 (4)	
O6	0.49935 (17)	−0.49559 (13)	0.19861 (18)	0.0799 (5)	
O7	0.75453 (16)	−0.46121 (13)	−0.12824 (17)	0.0778 (5)	
O8	0.81704 (14)	−0.30190 (14)	−0.18852 (14)	0.0655 (4)	
S1	0.72498 (4)	0.05824 (4)	−0.07550 (4)	0.04737 (13)	
S2	0.76812 (4)	−0.10241 (4)	−0.12039 (4)	0.04554 (12)	

### Atomic displacement parameters (Å^2^)

**Table:**

	*U*^11^	*U*^22^	*U*^33^	*U*^12^	*U*^13^	*U*^23^
C1	0.0357 (7)	0.0502 (9)	0.0413 (8)	−0.0104 (6)	0.0067 (6)	−0.0055 (7)
C2	0.0488 (9)	0.0382 (8)	0.0513 (9)	−0.0065 (7)	0.0143 (7)	−0.0080 (7)
C3	0.0412 (8)	0.0335 (7)	0.0499 (9)	−0.0008 (6)	0.0103 (7)	0.0043 (6)
C4	0.0359 (7)	0.0335 (7)	0.0390 (7)	−0.0070 (5)	0.0064 (6)	0.0041 (5)
C5	0.0407 (8)	0.0332 (7)	0.0467 (8)	−0.0027 (6)	0.0038 (6)	−0.0013 (6)
C6	0.0356 (7)	0.0427 (8)	0.0487 (9)	−0.0013 (6)	0.0020 (6)	0.0029 (7)
C7	0.0326 (7)	0.0360 (7)	0.0384 (7)	−0.0004 (5)	0.0012 (5)	0.0030 (5)
C8	0.0381 (7)	0.0320 (6)	0.0458 (8)	0.0035 (5)	−0.0016 (6)	−0.0028 (6)
C9	0.0325 (7)	0.0387 (7)	0.0360 (7)	0.0036 (5)	0.0004 (5)	−0.0076 (6)
C10	0.0318 (6)	0.0378 (7)	0.0331 (6)	−0.0037 (5)	0.0009 (5)	−0.0017 (5)
C11	0.0427 (8)	0.0309 (6)	0.0440 (8)	−0.0003 (6)	0.0077 (6)	−0.0032 (6)
C12	0.0383 (7)	0.0362 (7)	0.0414 (8)	0.0024 (6)	0.0097 (6)	−0.0032 (6)
N1	0.0498 (9)	0.0760 (12)	0.0524 (9)	−0.0147 (8)	−0.0006 (7)	−0.0107 (8)
N2	0.0671 (11)	0.0374 (8)	0.0714 (11)	0.0071 (7)	0.0062 (9)	0.0083 (7)
N3	0.0443 (8)	0.0449 (8)	0.0540 (9)	−0.0049 (6)	0.0050 (6)	0.0119 (6)
N4	0.0445 (8)	0.0499 (8)	0.0485 (8)	0.0045 (6)	0.0063 (6)	−0.0146 (6)
O1	0.0865 (13)	0.1028 (15)	0.0845 (13)	0.0076 (11)	−0.0384 (10)	−0.0111 (11)
O2	0.0828 (12)	0.0917 (12)	0.0701 (11)	−0.0154 (10)	0.0018 (9)	−0.0382 (9)
O3	0.196 (3)	0.0589 (11)	0.0986 (15)	0.0615 (14)	−0.0184 (16)	−0.0123 (10)
O4	0.0770 (11)	0.0504 (8)	0.0972 (13)	0.0045 (7)	−0.0282 (10)	0.0116 (8)
O5	0.0730 (10)	0.0733 (10)	0.0646 (9)	−0.0068 (8)	0.0304 (8)	0.0071 (8)
O6	0.0989 (13)	0.0415 (7)	0.1001 (13)	−0.0095 (8)	0.0274 (10)	0.0162 (8)
O7	0.0914 (12)	0.0486 (8)	0.0946 (13)	0.0047 (8)	0.0382 (10)	−0.0246 (8)
O8	0.0707 (9)	0.0673 (9)	0.0594 (8)	−0.0052 (7)	0.0297 (7)	−0.0162 (7)
S1	0.0535 (3)	0.0414 (2)	0.0469 (2)	−0.00851 (17)	−0.00634 (18)	0.00949 (16)
S2	0.0508 (2)	0.0483 (2)	0.0377 (2)	−0.01204 (17)	0.00926 (16)	−0.00253 (16)

### Geometric parameters (Å, º)

**Table:**

C1—C2	1.369 (3)	C9—C10	1.400 (2)
C1—C6	1.373 (2)	C9—N4	1.460 (2)
C1—N1	1.468 (2)	C10—C11	1.396 (2)
C2—C3	1.373 (3)	C10—S2	1.7723 (15)
C2—H2	0.9300	C11—C12	1.378 (2)
C3—C4	1.396 (2)	C11—H11	0.9300
C3—N2	1.463 (2)	C12—H12	0.9300
C4—C5	1.396 (2)	N1—O1	1.216 (3)
C4—S1	1.7738 (16)	N1—O2	1.219 (3)
C5—C6	1.377 (2)	N2—O4	1.207 (3)
C5—H5	0.9300	N2—O3	1.211 (3)
C6—H6	0.9300	N3—O5	1.215 (2)
C7—C8	1.371 (2)	N3—O6	1.215 (2)
C7—C12	1.373 (2)	N4—O7	1.211 (2)
C7—N3	1.463 (2)	N4—O8	1.218 (2)
C8—C9	1.376 (2)	S1—S2	2.0458 (7)
C8—H8	0.9300		
			
C2—C1—C6	122.07 (16)	C8—C9—N4	116.01 (14)
C2—C1—N1	118.61 (17)	C10—C9—N4	121.21 (14)
C6—C1—N1	119.31 (17)	C11—C10—C9	116.81 (13)
C1—C2—C3	117.81 (15)	C11—C10—S2	122.06 (12)
C1—C2—H2	121.1	C9—C10—S2	121.13 (11)
C3—C2—H2	121.1	C12—C11—C10	121.42 (14)
C2—C3—C4	122.93 (15)	C12—C11—H11	119.3
C2—C3—N2	116.33 (16)	C10—C11—H11	119.3
C4—C3—N2	120.74 (16)	C7—C12—C11	118.92 (14)
C3—C4—C5	116.76 (15)	C7—C12—H12	120.5
C3—C4—S1	121.68 (12)	C11—C12—H12	120.5
C5—C4—S1	121.54 (12)	O1—N1—O2	124.45 (19)
C6—C5—C4	121.21 (15)	O1—N1—C1	117.14 (19)
C6—C5—H5	119.4	O2—N1—C1	118.4 (2)
C4—C5—H5	119.4	O4—N2—O3	123.72 (19)
C1—C6—C5	119.21 (16)	O4—N2—C3	118.27 (17)
C1—C6—H6	120.4	O3—N2—C3	118.0 (2)
C5—C6—H6	120.4	O5—N3—O6	123.81 (17)
C8—C7—C12	122.49 (14)	O5—N3—C7	118.59 (15)
C8—C7—N3	118.38 (14)	O6—N3—C7	117.57 (16)
C12—C7—N3	119.13 (14)	O7—N4—O8	123.85 (16)
C7—C8—C9	117.59 (14)	O7—N4—C9	118.39 (16)
C7—C8—H8	121.2	O8—N4—C9	117.76 (15)
C9—C8—H8	121.2	C4—S1—S2	104.44 (6)
C8—C9—C10	122.78 (14)	C10—S2—S1	105.01 (5)
			
C6—C1—C2—C3	−0.7 (3)	N3—C7—C12—C11	179.17 (14)
N1—C1—C2—C3	−179.85 (15)	C10—C11—C12—C7	0.3 (2)
C1—C2—C3—C4	1.3 (2)	C2—C1—N1—O1	−175.60 (19)
C1—C2—C3—N2	−178.22 (15)	C6—C1—N1—O1	5.2 (3)
C2—C3—C4—C5	−0.9 (2)	C2—C1—N1—O2	2.9 (3)
N2—C3—C4—C5	178.56 (15)	C6—C1—N1—O2	−176.26 (18)
C2—C3—C4—S1	177.43 (13)	C2—C3—N2—O4	171.12 (18)
N2—C3—C4—S1	−3.1 (2)	C4—C3—N2—O4	−8.4 (3)
C3—C4—C5—C6	0.0 (2)	C2—C3—N2—O3	−7.9 (3)
S1—C4—C5—C6	−178.38 (12)	C4—C3—N2—O3	172.6 (2)
C2—C1—C6—C5	−0.2 (3)	C8—C7—N3—O5	173.98 (16)
N1—C1—C6—C5	178.95 (15)	C12—C7—N3—O5	−5.4 (2)
C4—C5—C6—C1	0.6 (2)	C8—C7—N3—O6	−4.1 (2)
C12—C7—C8—C9	0.1 (2)	C12—C7—N3—O6	176.51 (17)
N3—C7—C8—C9	−179.24 (13)	C8—C9—N4—O7	2.6 (2)
C7—C8—C9—C10	−0.2 (2)	C10—C9—N4—O7	−177.71 (17)
C7—C8—C9—N4	179.53 (14)	C8—C9—N4—O8	−177.67 (16)
C8—C9—C10—C11	0.3 (2)	C10—C9—N4—O8	2.1 (2)
N4—C9—C10—C11	−179.36 (14)	C3—C4—S1—S2	178.71 (12)
C8—C9—C10—S2	179.38 (12)	C5—C4—S1—S2	−3.03 (14)
N4—C9—C10—S2	−0.3 (2)	C11—C10—S2—S1	−4.57 (14)
C9—C10—C11—C12	−0.4 (2)	C9—C10—S2—S1	176.45 (11)
S2—C10—C11—C12	−179.43 (13)	C4—S1—S2—C10	80.78 (7)
C8—C7—C12—C11	−0.2 (2)		

### Hydrogen-bond geometry (Å, º)

**Table:**

*D*—H···*A*	*D*—H	H···*A*	*D*···*A*	*D*—H···*A*
C5—H5···O2^i^	0.93	2.54	3.341 (3)	144
C8—H8···O3^ii^	0.93	2.60	3.403 (2)	144
C12—H12···O6^iii^	0.93	2.42	3.139 (2)	134

Symmetry codes: (i) −*x*+2, *y*−1/2, −*z*+1/2; (ii) *x*, *y*−1, *z*; (iii) −*x*+1, *y*+1/2, −*z*+1/2.
